# Cisplatin eligibility in the neoadjuvant setting of patients with muscle-invasive bladder cancer undergoing radical cystectomy

**DOI:** 10.1093/oncolo/oyae160

**Published:** 2024-07-02

**Authors:** Renate Pichler, Josef Fritz, Andrea Mari, Anna Cadenar, Markus von Deimling, Gautier Marcq, Francesco del Giudice, Costantino Leonardo, Eugenio Bologna, Keiichiro Mori, Rana Tahbaz, Maria De Santis, Tobias Klatte, Barbara Erber, Felizian Lackner, Andreas Kronbichler, Andreas Seeber, Margit Fisch, Marco Moschini, Benjamin Pradere, Laura S Mertens

**Affiliations:** Department of Urology, Comprehensive Cancer Center Innsbruck (CCCI), Medical University of Innsbruck, Innsbruck 6020, Austria; Institute of Medical Statistics and Informatics, Medical University of Innsbruck, Innsbruck 6020, Austria; Department of Experimental and Clinical Medicine, University of Florence, Unit of Oncologic Minimally-Invasive Urology and Andrology, Careggi Hospital, Florence, Italy; Department of Experimental and Clinical Medicine, University of Florence, Unit of Oncologic Minimally-Invasive Urology and Andrology, Careggi Hospital, Florence, Italy; Department of Urology, University Medical Center Hamburg-Eppendorf, Hamburg 20249, Germany; Department of Urology, Claude Huriez Hospital, CHU Lille, Lille 59037, France; Department of Maternal Infant and Urologic Sciences, “Sapienza” University of Rome, Policlinico Umberto I Hospital, Rome 00185, Italy; Department of Maternal Infant and Urologic Sciences, “Sapienza” University of Rome, Policlinico Umberto I Hospital, Rome 00185, Italy; Department of Maternal Infant and Urologic Sciences, “Sapienza” University of Rome, Policlinico Umberto I Hospital, Rome 00185, Italy; Department of Urology, The Jikei University School of Medicine, 105-8461 Tokyo, Japan; Department of Urology, Charité Universitätsmedizin Berlin, Berlin 10117, Germany; Department of Urology, Charité Universitätsmedizin Berlin, Berlin 10117, Germany; Department of Urology, Medical University Vienna, Vienna 1090, Austria; Department of Urology, Charité Universitätsmedizin Berlin, Berlin 10117, Germany; Department of Urology, Charité Universitätsmedizin Berlin, Berlin 10117, Germany; Department of Urology, Comprehensive Cancer Center Innsbruck (CCCI), Medical University of Innsbruck, Innsbruck 6020, Austria; Department of Medicine, University of Cambridge, Cambridge CB2 0QQ, United Kingdom; Department of Internal Medicine IV, Medical University of Innsbruck, Innsbruck 6020, Austria; Department of Hematology and Oncology, Comprehensive Cancer Center Innsbruck, Medical University of Innsbruck, Innsbruck 6020, Austria; Department of Urology, University Medical Center Hamburg-Eppendorf, Hamburg 20249, Germany; Department of Urology, IRCCS Ospedale San Raffaele and Vita-Salute San Raffaele University, Milan 20132, Italy; Department of Urology, La Croix du Sud Hospital, 31130 Quint-Fonsegrives, France; Department of Urology, The Netherlands Cancer Institute, Antoni van Leeuwenhoek Hospital, Amsterdam 1006, The Netherlands

**Keywords:** muscle-invasive bladder cancer, radical cystectomy, neoadjuvant chemotherapy, cisplatin eligibility, kidney function, creatinine clearance

## Abstract

**Background:**

To examine the agreement of different calculated estimated glomerular filtration rate (eGFR) formulas and measured creatinine clearance (CrCI) at the primary diagnosis of muscle-invasive bladder cancer (MIBC).

**Materials and Methods:**

We performed a multicenter analysis of patients with MIBC, treated with cisplatin-based neoadjuvant chemotherapy (NAC) and radical cystectomy (RC), or with RC alone, between 2011 and 2021. Baseline eGFR was computed using 4 calculated serum equations including Cockcroft-Gault (CG), MDRD, CKD-EPI 2009, and race-free CKD-EPI 2021. To examine the association between calculated eGFR and measured CrCI, subgroup analyses were performed among patients in whom measured 24-hour urine CrCl was determined. Cisplatin-ineligibility was defined as CrCI and/or eGFR < 60 mL/minute per 1.73 m^2^.

**Results:**

Of 956 patients, 30.0%, 33.3%, 31.9%, and 27.7% were found to be cisplatin-ineligible by the CG, MDRD, CKD-EPI, and race-free CKD-EPI equations (*P* = .052). The concordance between calculated eGFR formulas was rated substantial (Cohen’s kappa (*k*): 0.66-0.95). Among the subgroup (*n* = 245) with measured CrCl, 37 (15.1%) patients had a CrCI less than 60 mL/minute. Concordance between measured CrCl and calculated eGFR was poor (*ĸ*: 0.29-0.40). All calculated eGFR formulas markedly underestimated the measured CrCI. Specifically, 78%-87.5% of patients with a calculated eGFR between 40 and 59 mL/minute exhibited a measured CrCI ≥ 60 mL/minute.

**Conclusions:**

Comparing calculated eGFR formulas, similar percentages of patients with MIBC were deemed cisplatin-ineligible. However, a significant number of patients could be upgraded by being cisplatin-fit based on measured CrCI, particularly when the calculated eGFR was falling within the gray range of 40-59 mL/minute.

## Teaser

This study examined the agreement of different calculated estimated glomerular filtration rate formulas and measured creatinine clearance at the primary diagnosis of muscle-invasive bladder cancer.

## Graphical Abstract

**Graphical Abstract:**
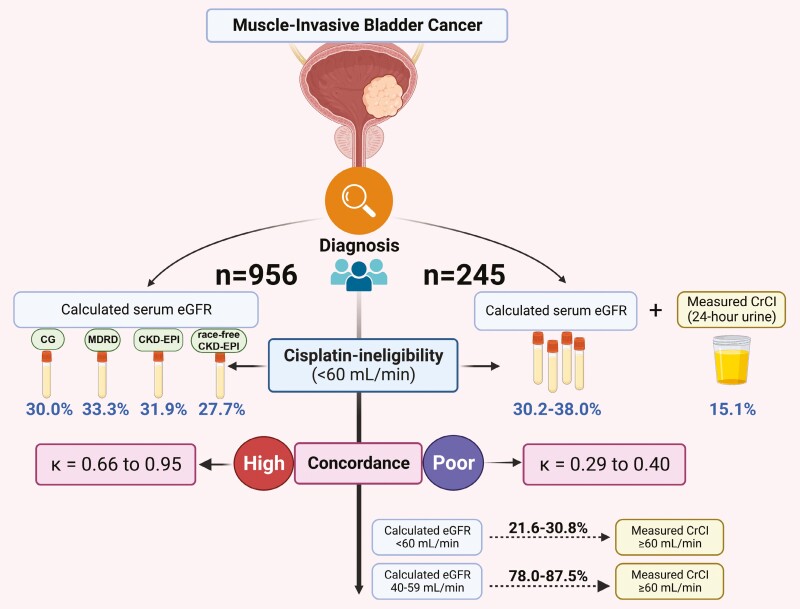

**Implications for practice**

All investigated formulas for eGFR calculation result in comparable percentages of cisplatin-unfit patients with MIBC. However, a significant number of patients could be upgraded by being cisplatin-fit based on measured CrCI. In patients with calculated eGFR falling within the 40-59 mL/minute range, up to 87.5% exhibited a measured CrCl ≥ 60 mL/minute.

## Introduction

The standard treatment for patients with localized or locally advanced muscle-invasive bladder cancer (MIBC) is radical cystectomy (RC) with pelvic lymph node dissection,^1^ preceded by cisplatin-based neoadjuvant chemotherapy (NAC).^2^ Meta-analyses of randomized trials have demonstrated an absolute overall survival improvement by 5%-8% at 5 years for cisplatin-based NAC followed by RC in comparison with RC alone.^3^ However, cisplatin-ineligibility in MIBC is caused by multiple factors, such as renal dysfunction, poor performance status, neuropathy, hearing loss, and cardiovascular dysfunction that may exclude nearly 50% of patients undergoing RC from receiving cisplatin-based NAC.^4^ While various objective parameters influence eligibility for cisplatin-based NAC, it is crucial to emphasize the necessity of adequate kidney function owing to cisplatin’s nephrotoxic potential.

In metastatic disease, cisplatin ineligibility is specifically defined by the Galsky criteria including a WHO or ECOG performance status (PS) of 2 or Karnofsky PS of 60%-70%, peripheral neuropathy (CTCAE v4 grade ≥ 2), audiometric hearing loss (CTCAE v4 grade ≥ 2), NYHA Class III heart failure and a creatinine clearance (calculated or measured) < 60 mL/minute.^5^ Calculated eGFR formulas may show discrepancies and might underestimate the actual creatinine clearance (CrCl), therefore affecting the eligibility for cisplatin-based chemotherapy.^6,7^ Thus, measured CrCI has been suggested to be a better index to identify candidates for cisplatin-based chemotherapy.^8^ Although a CrCI > 60 mL/minute is frequently used to characterize cisplatin-eligible patients also in the neoadjuvant setting, there is no clear guideline-recommendation about the best method used to determine kidney function.

The efficacy of calculated eGFR formulas in determining cisplatin-eligibility in the neoadjuvant setting has only been explored in a single trial, showing a similar proportion of cisplatin-eligible patients between 70% and 75%.^9^ However, diverse definitions for adequate kidney function in the context of cisplatin-based chemotherapy may lead to undertreatment of MIBC by inappropriately excluding patients from chemotherapy.^10-13^ To this end, we investigated the impact of serum and urine equations for estimating kidney function on the eligibility for cisplatin-based NAC in patients with MIBC, in a large retrospective multicenter analysis.

## Patients and methods

### Study design and patient selection

We performed a retrospective analysis of individual patient data originating from our international multicenter urothelial cancer collaboration group. Patient data and treatment information of patients with cT2-4aN0M0 urothelial carcinoma of the bladder treated consecutively with RC, with or without NAC, between 2011 and 2021 were retrospectively collected. Each patient included in this retrospective study had to have a serum creatinine level (±urine CrCI) at the time of initial diagnosis of MIBC.

The eGFR at the primary diagnosis of MIBC, defined as prior to RC or NAC, was determined using 4 different serum equations, namely Cockcroft-Gault (CG), Modification of Diet in Renal Disease (MDRD), Chronic Kidney Disease Epidemiology Collaboration (CKD-EPI) 2009, and race-free CKD-EPI 2021.^14-17^ The selection of these specific equations in the study was based on their established accuracy, widespread use, and ability to provide reliable estimates of kidney function across diverse populations. Furthermore, in a subset of patients, the measured CrCI was determined under controlled conditions, and assessed through a 24-hour urine collection (with indication of the exact urine volume) and measurement of urine creatinine, and serum creatinine during the inpatient stay. Standard methodologies were used to generate estimates of calculated and measured CrCI as shown in Supplementary Table S1. Cisplatin ineligibility was defined based on the most commonly used threshold value in clinical settings, namely CrCI and/or eGFR < 60 mL/minute per 1.73m^2^.^5^ A detailed overview of the study design is shown in Supplementary Figure S1.

### Statistical analysis

The agreement and concordance between measures of CrCl (CG, MDRD, CKD-EPI, race-free CKD-EPI, and 24 hour urine) were evaluated using Bland-Altman plots,^18^ calculation of systematic differences between measurements (ie, fixed bias) and Pearson correlation coefficients for CrCl on a continuous scale. Additionally, the agreement between CrCl measurements as a dichotomized variable, with a cutoff value of 60 mL/minute per 1.73 m^2^, was evaluated using simple percent agreement calculation and Cohen’s kappa *κ*. Given that the decision on cisplatin eligibility is based on this dichotomized CrCl version, it was crucial to focus our analyses on the dichotomized variables. Subgroup analyses were performed to identify groups exhibiting particularly high or low agreement between measurements. Probabilities for a measured CrCl ≥ 60 mL/minute per 1.73 m^2^, given a specific calculated CrCl value, were derived from logistic regression models on log-transformed calculated CrCl values. Including interaction terms in these models, the potential of clinical parameters as effect moderators of these probabilities was evaluated using likelihood-ratio tests. Statistical analyses were conducted in R, version 4.0.5.^19^ All statistical tests were 2-sided at a significance level of 0.05. Figures were created with Biorender and GraphPad.

## Results

### Patient population

A total of 956 patients with MIBC who underwent RC were analyzed; 276 (28.9%) were treated with NAC followed by RC, and 680 (71.1%) with RC only. The median age was 70 years (range, 28 to 92 years), with 657 (68.7%) patients being 65 years or older. Twenty-three percent were female. Patient characteristics of the total cohort and stratified by NAC status are displayed in detail in Table 1.

**Table 1.** Descriptive statistics of clinicopathological characteristics of the total study patient cohort (*n* = 956) and stratified by neoadjuvant chemotherapy (NAC) status.

Parameters	Total	RC alone	RC + NAC	*P* value
	(*n* = 956)	(*n* = 680)	(*n* = 276)	
*Male sex*				
*n* (%)	740 (77.4%)	537 (79.0%)	203 (73.6%)	.0735
*Age, years*				
Mean (SD)	69.0 (10.4)	71.2 (9.8)	63.8 (9.8)	**<.001**
*Ethnicity, n (%)*				
White	931 (97.4%)	680 (100%)	251 (90.9%)	**<.001**
Black	1 (0.1%)	0 (0%)	1 (0.4%)	
Other	24 (2.5%)	0 (0%)	24 (8.7%)	
*BMI* ^1^ *, kg/m* ^*2*^				
Mean (SD)	25.7 (4.5)	25.6 (4.5)	26.0 (4.6)	.2968
Missing, *n* (%)	19 (1.9%)	12 (1.8%)	7 (2.5%)	
*ECOG* ^1^				
0	552 (68.3%)	377 (66.3%)	175 (73.2%)	.0568
≥1	256 (31.7%)	192 (33.7%)	64 (26.8%)	
Missing	148 (15.5%)	111 (16.3%)	37 (13.4%)	
*Smoking* ^1^				
Nonsmoker	219 (42.8%)	114 (40.6%)	105 (45.5%)	**.006**
Ex-smoker	130 (25.4%)	87 (30.9%)	43 (18.6%)	
Current smoker	163 (31.8%)	80 (28.5%)	83 (35.9%)	
Missing	444 (46.4%)	399 (58.7%)	45 (16.3%)	
*Prevalent diabetes* ^1^				
Yes	69 (12.2%)	38 (12.2%)	31 (13.4%)	.5342
Missing, *n* (%)	414 (43.3%)	369 (54.3%)	45 (16.3%)	
*MIBC*				
Primary	867 (90.7%)	623 (91.6%)	244 (88.4%)	.1401
Secondary	89 (9.3%)	57 (8.4%)	32 (11.6%)	
*Hydronephrosis at diagnosis* ^1^				
No	412 (77.6%)	197 (74.6%)	215 (80.5%)	.1104
Yes	119 (22.4%)	67 (25.4%)	52 (19.5%)	
Missing	425 (44.5%)	416 (61.2%)	9 (3.3%)	
*pT stage at RC*				
≤pT1	218 (22.8%)	103 (15.1%)	115 (41.7%)	**<.001**
pT2	239 (25.0%)	190 (27.9%)	49 (17.8%)	
pT3	351 (36.7%)	275 (40.4%)	76 (27.5%)	
pT4	140 (14.6%)	112 (16.5%)	28 (10.1%)	
Missing	8 (0.8%)	0 (0%)	8 (2.9%)	
*Number of LNs resected*				
Mean (SD)	18.3 (11.5)	16.6 (11.4)	22.7 (10.5)	**<.001**
Median (Q1, Q3)	17.0 (11.0, 24.0)	14.0 (9.0, 23.0)	22.0 (16.0, 28.0)	
*Number of pos. LNs*				
Mean (SD)	1.1 (2.9)	1.2 (3.0)	0.9 (2.4)	**.0391**
*Number of NAC cycles*				
Mean (SD)	—	—	3.2 (1.1)	—
Median (*Q*1, *Q*3)	—	—	4.0 (2.0, 4.0)	

*P*-values from Fisher’s exact tests for categorical variables, and Welch’s *t*-tests and Wilcoxon tests for numerical variables. Bold values show statistically significant *P* values.

^1^ *N* for total number of variables available to calculate prevalence.

Abbreviations: BMI, body mass index; ECOG, Eastern Cooperative Oncology Group; LN, lymph node MIBC, muscle invasive bladder cancer; NAC, neoadjuvant chemotherapy; RC, radical cystectomy; SD, standard deviation.

In the RC only group, the most common reasons why NAC was not administered were physician’s or patient’s preference (*n* = 397), impaired kidney function (*n* = 86), other reasons (*n* = 41), and not specified (*n* = 157). NAC schedules consisted of gemcitabine-cisplatin (Gem-Cis; 73.0%) and dose-dense methotrexate-vinblastine-doxorubicin hydrochloride-cisplatin (ddMVAC; 2.7%). Other NAC regimens included gemcitabine-carboplatin (Gem-Carbo; 24.3%). The median number of applicated NAC cycles was 4 (IQR, 2-4), respectively. In patients treated with NAC followed by RC, cisplatin-eligible patients had a better OS when compared with those who had a CrCI < 60 mL/minute (CG: *P* = .034; MDRD: *P* = .022; CKD-EPI: *P* = .014; CKD-EPI race-free: *P* = .083), respectively.

### Calculated eGFR formulas

Regarding kidney function, 30.0% (95% CI, 26.3%-34.0%), 33.3% (95% CI, 30.3%-36.3%), 31.9% (95% CI, 29.0%-34.9%), and 27.7% (95% CI, 25.0%-30.6%) of total patients were found to be cisplatin-ineligible by the calculated CG, MDRD, CKD-EPI and race-free CKD-EPI equations (*P* = .052), Table 2.

**Table 2.** Descriptive statistics of calculated eGFR and measured CrCI values of the total patient cohort.

eGFR formulas	Total
	(*N* = 956)
*CG*	
Missing^1^	403 (42.2%)
Mean (SD)	76.2 (31.9)
Median (Q1, Q3)	72.9 (55.4, 93.2)
Min, max	9.1 (255.6)
<40	65 (11.8%)
40-59	101 (18.3%)
≥60	387 (70.0%)
*MDRD*	
Mean (SD)	70.2 (23.9)
Median (*Q*1, *Q*3)	69.6 (53.2, 84.2)
Min, max	6.6 (180.2)
<40	94 (9.8%)
40-59	224 (23.4%)
≥60	638 (66.7%)
*CKD-EPI 2009*	
Mean (SD)	69.8 (21.6)
Median (Q1, Q3)	72.1 (54.3, 86.8)
Min, max	6.0 (127.4)
<40	95 (9.9%)
40-59	210 (22.0%)
≥60	651 (68.1%)
*CKD-EPI racefree 2021*	
Mean (SD)	73.9 (22.2)
Median (*Q*1, *Q*3)	76.6 (57.8, 91.9)
Min, max	6.5 (125.0)
<40	73 (7.6%)
40-59	192 (20.1%)
≥60	691 (72.3%)
*Measured CrCl*	
Missing	711 (74.4%)
Mean (SD)	91.0 (35.7)
Median (*Q*1, *Q*3)	86.8 (66.6, 113.6)
Min, max	11.4 (221.9)
<40	17 (6.9%)
40-59	20 (8.2%)
≥60	208 (84.9%)

^1^Due to missing information on weight.

**Figure 1.**
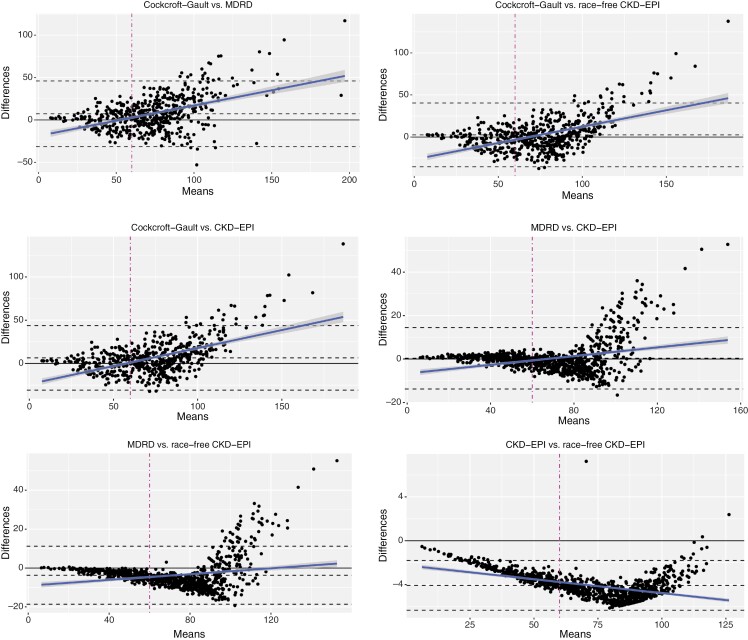
Bland-Altman plots for the agreement among the 4 calculated serum eGFR formulas. Limits of agreement are shown as top and bottom dashed lines, and the middle black line indicates the systematic differences between measurements (ie, fixed bias). The blue line is the regression fit of the differences in the means, and in gray shades, 95% confidence intervals are indicated. Values of the fixed bias were 7.26 mL/minute per 1.73 m^2^ for CG vs MDRD (ie, overestimation of CG over MDRD), 6.52 for CG vs CKD-EPI, 2.49 for CG vs race-free CKD-EPI, 0.35 for MDRD vs CKD-EPI, −3.72 for MDRD vs race-free CKD-EPI (ie, underestimation of MDRD over race-free CKD-EPI), and −4.07 for CKD-EPI vs race-free CKD-EPI. Importantly, agreement divergence (ie, differences on the *y*-axis being large) became more prevalent for higher eGFR values (ie, 100 and above), and values < 60 are much less affected. Note the scaling differences on x- and y-axes for the different panels.

Concordance between the calculated CrCI equations in determining cisplatin-eligibility was rated moderate to substantial, with *k* values between 0.66 and 0.95, Table 3. Focusing on specific patient subgroups, the agreement between calculated formulas remained moderate to substantial based on age, sex, Charlson Comorbidity Index score, and ethnicity. Overall correlation coefficients ranged from 0.79 to 0.96, indicating a strong correlation. Bland-Altman plots visualizing the agreement amongst calculated CrCI formulas are shown in Figure 1. Fixed bias (middle dashed black line) was in general relatively small (≤7.5) amongst all calculated CrCl formulas (largest for CG, though), and, importantly, agreement divergence became more prevalent only for eGFR values clearly higher than 60 mL/minute per 1.73m^2^, at which value the dichotomization into cisplatin eligibility (yes/no) takes place.

**Table 3.** Agreement and concordance rates, and correlation between calculated eGFR formulas.

eGFR formula	eGFR < 60 mL/minute	eGFR ≥ 60 mL/minute	Disagreement: eGFR ≥ 60, but < 60 in the reference % within those with < 60 mL/minute in the reference	Agreement: eGFR < 60 mL/minute both in the investigated formula and the reference % within those with < 60 mL/minute in the reference	Disagreement: eGFR < 60, but ≥ 60 in the reference % within those with ≥ 60 mL/minute in the reference	Agreement: eGFR ≥ 60 mL/minute both in the investigated formula and the reference % within those with ≥ 60 mL/minute in the reference	Kappa (95% CI)	Pearson rho (95% CI)
*Reference: race-free CKD-EPI*								
CG	166 (30.0%)	387 (70.0%)	24 (16.3%)	123 (83.7%)	43 (10.6%)	363 (89.4%)	0.70 (0.62-0.78)	0.80 (0.77-0.83)
MDRD	318 (33.3%)	638 (66.7%)	0 (0%)	265 (100%)	53 (7.7%)	638 (92.3%)	0.87 (0.81-0.93)	0.95 (0.94-0.95)
CKD-EPI	305 (31.9%)	651 (68.1%)	0 (0%)	265 (100%)	40 (5.8%)	651 (94.2%)	0.90 (0.84-0.96)	1.00 (1.00-1.00)
*Reference: CKD-EPI*								
CG	166 (30.0%)	387 (70.0%)	41 (23.2%)	136 (76.8%)	30 (8.0%)	346 (92.0%)	0.70 (0.62-0.78)	0.81 (0.78-0.84)
MDRD	318 (33.3%)	638 (66.7%)	3 (1%)	302 (99.0%)	16 (2.5%)	635 (97.5%)	0.95 (0.89-1.02)	0.95 (0.95-0.96)
race-free CKD-EPI	265 (27.7%)	691 (72.3%)	40 (13.1%)	265 (86.9%)	0 (0%)	651 (100%)	0.90 (0.84-0.96)	1.00 (1.00-1.00)
*Reference: MDRD*								
CG	166 (30.0%)	387 (70.0%)	51 (27.4%)	135 (72.6%)	31 (8.4%)	336 (91.6%)	0.66 (0.58-0.74)	0.79 (0.76-0.82)
CKD-EPI	305 (31.9%)	651 (68.1%)	16 (5.0%)	302 (95.0%)	3 (0.5%)	635 (99.5%)	0.95 (0.89-1.02)	0.95 (0.95-0.96)
CKD-EPIrace-free	265 (27.7%)	691 (72.3%)	53 (16.7%)	265 (83.3%)	0 (0%)	638 (100.0%)	0.87 (0.81-0.93)	0.95 (0.94-0.95)
*Reference: CG*								
MDRD	318 (33.3%)	638 (66.7%)	31 (18.7%)	135 (81.3%)	51 (13.2%)	336 (86.8%)	0.66 (0.58-0.74)	0.79 (0.76-0.82)
CKD-EPI	305 (31.9%)	651 (68.1%)	30 (18.1%)	136 (81.9%)	41 (10.6%)	346 (89.4%)	0.70 (0.62-0.78)	0.81 (0.78-0.84)
CKD-EPI race-free	265 (27.7%)	691 (72.3%)	43 (25.9%)	123 (74.1%)	24 (6.2%)	363 (93.8%)	0.70 (0.62-0.78)	0.80 (0.77-0.83)

### Calculated eGFR and measured CrCI

Among a subgroup of 245 patients, the median measured CrCI was 86.8 mL/minute (range, 11.4-221.9 mL/minute). There were significant differences in terms of the specific study center and other variables, in particular smoking status, age, ethnicity, presence of hydronephrosis, application of NAC, number of resected and positive lymph nodes, and final tumor histology at RC between those patients with vs. without measured CrCI, Table 4.

**Table 4.** Descriptive statistics of clinicopathological characteristics based on the presence of measured creatinine clearance (CrCI).

Parameters	Measured CrCI	No measured CrCI	*P* value
	(*N* = 245)	(*N* = 711)	
*Center*			
Sapienza Rome	33 (13.5%	55 (7.7%)	**<.001**
University Florence	11 (4.5%)	402 (56.5%)	
Medical University Innsbruck	152 (62%)	0 (0%)	
Jikei	22 (9%)	0 (0%)	
NKI Amsterdam	16 (6.5%)	93 (13.1%)	
University Hospital Charite Berlin	11 (4.5%)	4 (0.6%)	
UKE Hamburg Eppendorf	0 (0%)	157 (22.1%)	
*Male sex*			
n (%)	138 (74.4%)	557 (78.3%)	.250
*Age, years*			
Mean (SD)	67.5 (9.9)	69.6 (10.5)	**.0059**
*Ethnicity, n (%)*			
White	223 (91.0%)	708 (99.6%)	**<.001**
Black	0 (0%)	1 (0.1%)	
Other	22 (9.0%)	2 (0.3%)	
*BMI* ^1^ *, kg/m* ^*2*^			
Mean (SD)	25.9 (4.3)	25.7 (4.6)	.5096
Missing, n (%)	1 (0.4%)	18 (2.5%)	
*ECOG* ^1^			
0	163 (69.7%)	389 (67.8%)	.6179
≥1	71 (30.3%)	185 (32.2%)	
Missing	11 (4.5%)	137 (19.3%)	
*Smoking* ^1^			
Non-smoker	140 (60.1%)	79 (28.3%)	**<.001**
Ex-smoker	53 (22.7%)	110 (39.4%)	
Current smoker	40 (17.2%)	90 (32.3%)	
Missing	12 (4.9%)	432 (60.8%)	
*Prevalent diabetes* ^1^			
Yes	26 (11.2%)	43 (13.9%)	.3873
Missing, *n* (%)	12 (4.9%)	402 (56.5%)	
*MIBC*			
Primary	218 (89%)	649 (91.3%)	.3078
Secondary	27 (11%)	62 (8.7%)	
*Hydronephrosis at diagnosis* ^1^			
No	198 (80.8%)	214 (74.9%)	**.007**
Yes	47 (19.2%)	72 (25.1%)	
Missing	0 (0%)	425 (59.8%)	
*pT stage at RC* ^1^			
≤pT1	82 (33.6%)	136 (19.2%)	**<.001**
pT2	57 (23.4%)	182 (25.7%)	
pT3	72 (29.5%)	279 (39.4%)	
pT4	33 (13.5%)	111 (15.7%)	
Missing	1 (0.4%)	3 (0.4%)	
*NAC application*			
Yes	140 (57.1%)	136 (19.1%)	**<.001**
*Number of pos. LNs resected*			
Mean (SD)	0.9 (2.7)	1.1 (2.9)	**.0023**
Median (Q1, Q3)	0.0 (0.0, 0.0)	0.0 (0.0, 1.0)	
*Number of resected LNs*			
Mean (SD)	22.1 (11.1)	17.1 (11.4)	**<.001**
Median (Q1, Q3)	22.0 (14, 28)	15.0 (10, 23)	
*CG*			
<60 mL/minute	84 (36.1%)	82 (25.6%)	**.0087**
≥60 mL/minute	149 (63.9%)	238 (74.4%)	
Missing	12 (4.9%)	391 (55.0%)	
*MDRD*			
<60 mL/minute	93 (38%)	225 (31.6%)	.0835
≥ 60 mL/minute	152 (62%)	486 (68.4%)	
*CKD-EPI 2009*			
<60 mL/minute	85 (34.7%)	220 (30.9%)	.3016
≥60 mL/minute	160 (65.3%)	491 (69.1%)	
*CKD-EPI 2021*			
<60 mL/minute	74 (30.2%)	191 (26.9%)	.3214
≥60 mL/minute	171 (69.8%)	520 (73.1%)	

*P*-values from Fisher’s exact tests for categorical variables, and Welch’s *t*-tests and Wilcoxon tests for numerical variables. Bold values show statistically significant *P* values.

^1^ *N* for total number of variables available to calculate prevalence.

Abbreviations: BMI, body mass index; ECOG, Eastern Cooperative Oncology Group; LN, lymph node; MIBC, muscle invasive bladder cancer; NAC, neoadjuvant chemotherapy; RC, radical cystectomy; SD, standard deviation.

**Figure 2.**
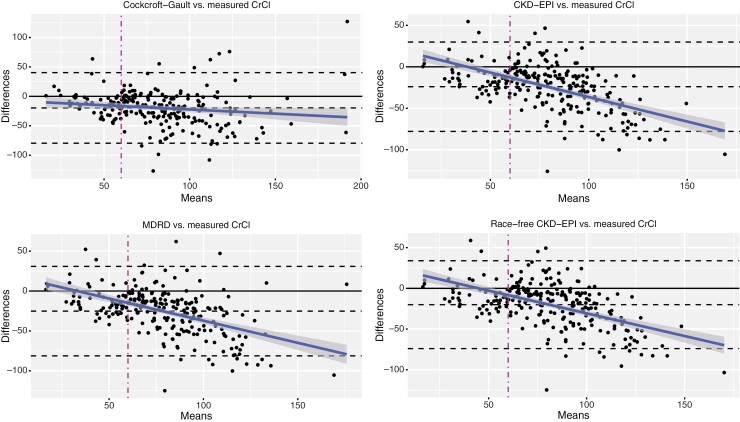
Bland-Altman plots for the agreement among calculated eGFR and measured CrCI formulas. Limits of agreement are shown as top and bottom dashed lines, and the middle black line indicates the systematic differences between measurements (ie, fixed bias). The blue line is the regression fit of the differences in the means, and in gray shades, 95% CIs are indicated. Fixed bias was −19.62, −25.18, −24.06, and −20.08 mL/minute per 1.73 m^2^ for CG, MDRD, CKD-EPI, and race-free CKD-EPI vs measured CrCl, respectively. As seen by the negative regression slopes, underestimation of the measured CrCl increased the higher CrCl values became. Note the scaling differences on x- and y-axes for the different panels.

At baseline, 37 (15.1%; 95% CI, 11.2%-20.1%) patients had a measured CrCI less than 60 mL/minute. In contrast, 30.2%-38% of the same cohort had a calculated CrCI < 60 mL/minute (*P* < .001). Concordance between measured and calculated CrCI was poor (range of *ĸ* = 0.29-0.40), Table 5, mainly because among patients with a measured CrCI ≥ 60 mL/minute, still 29.3% (CG), 30.8% (MDRD), 26.9% (CKD-EPI), and 21.6% (race-free CKD-EPI) had a calculated CrCI < 60 mL/minute. The overall correlation coefficients between calculated and measured CrCI ranged from 0.60 to 0.64, indicating only a moderate correlation between all values of calculated and measured CrCI. These findings were also confirmed by the Bland-Altman plots in Figure 2, showing that calculated CrCl formulas underestimate measured CrCl by −19.62, −25.18, −24.06, and −20.08 mL/minute per 1.73 m^2^ for CG, MDRD, CKD-EPI, and race-free CKD-EPI, bias values much higher than among the calculated CrCl formulas themselves. The bias got worse the higher the CrCl values were (negative slope of the regression lines).

**Table 5.** Agreement and concordance rates, and correlation between calculated eGFR formulas and measured CrCl.

eGFR formula	eGFR <60 mL/minute	eGFR ≥ 60 mL/minute	Disagreement: eGFR ≥ 60, but < 60 measured CrCl % within those with measured CrCl < 60 mL/minute	Agreement: eGFR < 60 mL/minute both in the investigated formula and measured CrCl % within those with measured CrCl < 60 mL/minute	Disagreement: eGFR < 60, but ≥ 60 measured CrCl % within those with measured CrCl ≥ 60 mL/minute	Agreement: eGFR ≥ 60 mL/minute both in the investigated formula and measured CrCl % within those with measured CrCl ≥ 60 mL/minute	Kappa (95% CI)	Pearson rho (95% CI)
CG	84 (36.1%)	149 (63.9%)	9 (25.7%)	26 (74.3%)	58 (29.3%)	140 (70.7%)	0.29 (0.18-0.39)	0.60 (0.51-0.67)
MDRD	93 (38.0%)	152 (62.0%)	8 (21.6%)	29 (78.4%)	64 (30.8%)	144 (69.2%)	0.29 (0.19-0.40)	0.60 (0.51-0.67)
CKD-EPI	85 (34.7%)	160 (65.3%)	8 (21.6%)	29 (78.4%)	56 (26.9%)	152 (73.1%)	0.34 (0.23-0.44)	0.64 (0.56-0.71)
CKD-EPI race-free	74 (30.2%)	171 (69.8%)	8 (21.6%)	29 (78.4%)	45 (21.6%)	163 (78.4%)	0.40 (0.29-0.52)	0.64 (0.56-0.71)

**Figure 3.**
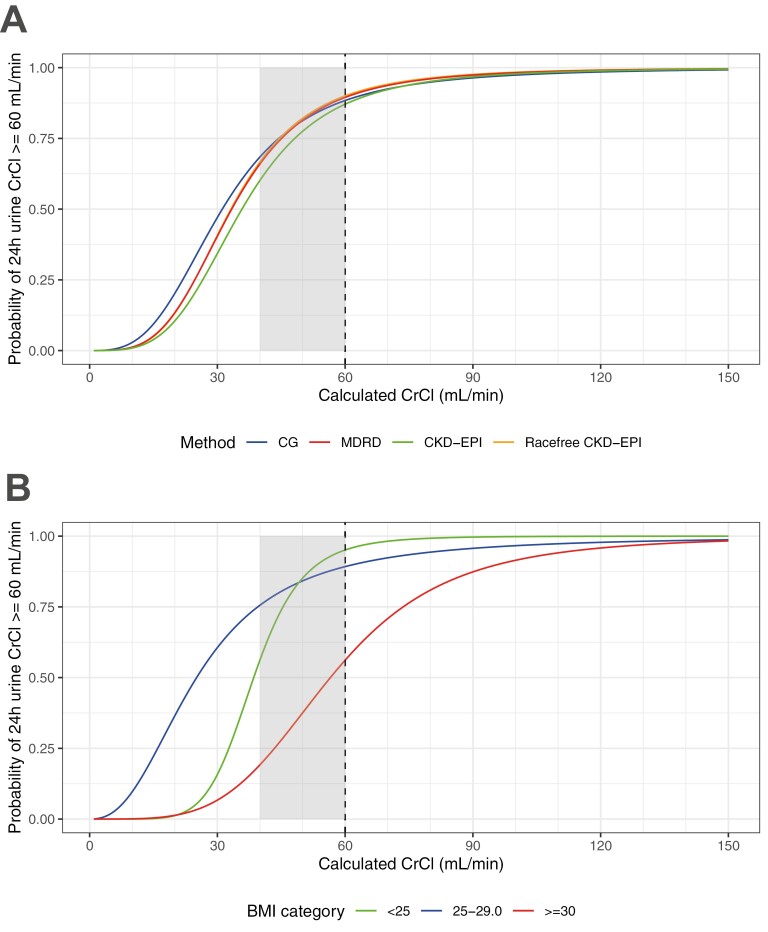
Probabilities of being cisplatin-eligible based on measured CrCl (urine CrCl ≥ 60 mL/minute) as a function of calculated eGFR values, for (A) different calculated eGFR formulas (MDRD, CG, CKD-EPI, and race-free CKD-EPI), and (B) BMI subgroups for the CG formula (the only clinical parameter and formula showing effect modification). Probabilities are derived from logistic regression models on log-transformed calculated CrCl values. *Note*: The orange curve of race-free CKD-EPI is nearly completely overlaid by the red MDRD curve and therefore hard to visually discern.

In patients with a calculated eGFR in the gray range between 40 and 59 mL/minute, the percentage of measured CrCI ≥ 60 mL/minute was 87.5% (CG), 86.2% (MDRD), 83.6% (CKD-EPI), and 78.0% (race-free CKD-EPI), Supplementary Table S2. Conversely, in case of a calculated eGFR of ≥60 mL/minute, the measured CrCl was also ≥60 mL/minute in more than 94% for all 4 measurement methods. The probability scores of logistic regression models displayed in Figure 3A corroborate this finding, by showing how likely it is, given a specific calculated CrCl value, that the measured CrCl value is ≥60 mL/minute. The curves for all 4 estimation methods cross the 50% mark well below 60 mL/minute, demonstrating the underestimation of measured CrCl values by the calculated serum formulas. Specifically, for a calculated CrCI of 40 mL/minute, the probability of a measured CrCl value ≥60 mL/minute is more than 50%, irrespective of the calculated CrCI formula. For a calculated CrCI of 50 mL/minute, the probability that the measured CrCI is ≥60 mL/minute is even higher, namely >75%. Focusing on clinical parameters, significant differences in these probability scores were only observed for subgroups of BMI using the CG formula (*P*_interaction_ = .003). Specifically, in obese patients (BMI ≥ 30 kg/m^2^), measured CrCI was much less underestimated compared to normal weight and overweight patients, Figure 3B. All other clinical parameters did not significantly modify the probability scores for all other formulas, Supplementary Table S3.

## Discussion

We hereby present the largest multicenter analysis, evaluating the impact of different serum and urine equation formulas for the assessment of kidney function^20^ in relation to NAC eligibility. Accurate patient selection for cisplatin-based NAC, based on the true kidney function, helps to avoid undertreatment in localized or locally advanced MIBC, in which NAC provides a survival benefit of 5%-8%, respectively.^3^ There are no precise eligibility criteria or guideline recommendations regarding NAC in MIBC that define (1) which serum eGFR formula is the most accurate method to assess true kidney function and (2) which role the measured CrCI plays in comparison to the calculated eGFR formulas. Thus, the optimal equation for estimating kidney function in patients with MIBC remains unclear and warrants further evaluation.

The measured CrCI formula is a traditional approach used to obtain the CrCI from a 24-hour urine collection. However, the clinical application of measured CrCI is low due to issues of convenience, compliance, practicability, collection failure, and costs.^21,22^ A strong correlation between measured and calculated CrCI was found in various tumors.^23,24^ However, studies have not adequately investigated the accuracy between measured and calculated CrCl in relation to bladder cancer, although these patients in particular are characterized by older age which is often associated with reduced eGFR.^2,4^ In metastatic disease, a retrospective study by Raj et al demonstrated a high discordance rate between calculated and measured CrCI formulas.^7^ Our results underscore this finding also in MIBC showing a poor concordance between measured CrCl and calculated eGFR (range of *ĸ* = 0.29-0.40). In our study, up to 31% of patients with calculated CrCI less than 60 mL/minute would have been eligible for cisplatin-based NAC by evaluation of measured CrCI. The underestimation of measured CrCI by calculated eGFR formulas is a complex issue with multiple contributing factors. Population characteristics can affect creatinine production and clearance, leading to discrepancies. Inherent limitations of eGFR formulas, such as their reliance on creatinine as a filtration marker, further add to the challenge. Additionally, factors like medication use and renal tubular secretion can affect eGFR accuracy. Understanding and addressing these variables are crucial for improving the precision of eGFR estimation and its clinical implications in patient care.

Methods for calculation of estimated CrCI by mathematical formulas vary significantly.^25^ According to the routine clinical use of calculated estimates of kidney function based on their reliability and reproducibility, the International Society of Geriatric Oncology Task Force on Renal Safety in the Elderly endorses calculated serum formulas for estimating kidney function.^26^ In addition to the CG equation,^17^ alternatives include the MDRD equation,^16^ the CKD-EPI equation introduced in 2009,^15^ and most recently, the race-free CKD-EPI formula 2021.^14^ Focusing on bladder cancer, there are first indications that individually calculated CrCI formulas show a heterogeneous accuracy in the determination of the cisplatin eligibility at a cutoff >60 mL/minute. In detail, Tsao et al found that the CKD-EPI equation was more likely to deem patients with locally advanced/metastatic bladder cancer (T2-T4, N+, or metastatic disease) cisplatin-fit as compared to the CG equation.^6^ In line with these findings, Horn et al demonstrated a good correlation between the MDRD and CKD-EPI equation (*r*^2^ = 0.92), whereas CG tended to underestimate GFR compared to MDRD and CKD-EPI in advanced urothelial cancer.^27^ Depending on the different calculated formula used, 12%-44% of the same cohort had a CrCI < 60 mL/minute representing strong variations in determining cisplatin eligibility in metastatic disease.^7^ Currently, only one study focused on the neoadjuvant setting of cisplatin-eligibility in bladder cancer, including 126 patients with MIBC. These findings suggest that the CG, MDRD, and CKD-EPI equations did not yield significant differences in cisplatin eligibility. The proportion of patients defined as cisplatin-eligible was 71% (CG), 70% (CKD-EPI), and 75% (MDRD), respectively.^9^ Our results corroborate these findings demonstrating a substantial concordance between calculated CrCI equations (*k* values: 0.66-0.95). The rate of cisplatin eligibility of each calculated CrCI formula was very similar with 70% (CG), 68.1% (CKD-EPI), 72.3% (race-free CKD-EPI), and 66.7% (MDRD), respectively. Therefore, we suggest that all calculated CrCI formulas can be used to determine kidney function for cisplatin eligibility in MIBC.

Thus, in clinical practice, it may not be necessary to routinely determine measured CrCI in every patient with MIBC. However, our results show that calculated CrCl values and this applies to all 4 investigated serum formulas, in general underestimate measured CrCl. Probability score analyses suggest that a specific subgroup of patients could benefit from additional assessment of measured CrCI. Specifically, patients falling within the “gray zone” with a calculated eGFR of 40-59 mL/minute could potentially be reclassified as cisplatin-eligible by using measured CrCl. According to our model, more than 50% and more than 75% of patients with a calculated CrCl of 40 mL/minute and 50 mL/minute, respectively, are incorrectly classified as cisplatin-ineligible. In detail, up to 87.5% of patients with a calculated CrCI between 40 and 59 mL/minute would have been cisplatin-eligible by evaluation of measured CrCI using a cutoff of 60 mL/minute. Importantly, these particular patients would either be subjected to upfront RC based on the assumption of impaired kidney function or receive cisplatin using alternative split-dose schedules. Currently, there are no prospective randomized trials comparing conventional cisplatin dosing with split-dose cisplatin in terms of oncological outcomes.^28,29^ Therefore, the exclusion of patients with a calculated eGFR of 40-59 mL/minute from cisplatin-based NAC, resulting in potential undertreatment, could be mitigated by incorporating measured CrCl. In our study, the likelihood of measuring a patient’s CrCl strongly depended on the study center, and the subgroup of patients with measured CrCI demonstrated higher rates of non-smokers (60.1% vs 28.3%), NAC application (57.1% vs 19.1%), and complete responders (≤pT1) at final histology in RC specimens (33.6% vs 19.2%) when compared with those without measured CrCl.

Limitations of this study include its retrospective design. Another limitation is the underrepresentation of Black patients in this cohort and the relatively small number of women. Hence, it cannot be guaranteed whether our findings are generalizable to these groups. More accurate measurement of eGFR as compared to 24-hour urine CrCI has not been used in our study, and in cases with uncertainty test methods such as iohexol plasma clearance, urinary inulin clearance, or ^99m^Tc DTPA clearance might be used to properly estimate eGFR.^30^

## Conclusions

Our study shows that all investigated formulas for eGFR calculation result in comparable percentages of cisplatin-unfit patients with MIBC. However, a significant number of patients could be upgraded by being cisplatin-fit based on measured CrCI. Depending on the specific calculated eGFR equation used, up to 31% of patients eligible for NAC based on measured CrCI would be classified as cisplatin-unfit. Whereas routine measured CrCl determination is not justified for all patients with MIBC, it is recommended to assess measured CrCl in patients with calculated eGFR falling within the 40-59 mL/minute range, as our models show that up to 87.5% of these patients exhibited a measured CrCl ≥ 60 mL/minute.

## Supplementary material

Supplementary material is available at *The Oncologist* online.

oyae160_suppl_Supplementary_Tables_1-3_Figures_1

## Data Availability

The data underlying this article will be shared on reasonable request to the corresponding author.
