# Supplementary material for: Cisplatin eligibility in the neoadjuvant setting of patients with muscle-invasive bladder cancer undergoing radical cystectomy
Source: Oncologist. 2024 Jul 2;29(11):e1511–22. doi: 10.1093/oncolo/oyae160 (PMC11546640; doi:10.1093/oncolo/oyae160)
Supplement: oyae160_suppl_Supplementary_Tables_1-3_Figures_1 [file oyae160_suppl_supplementary_tables_1-3_figures_1.pdf]

Supplementary material

Suppl. Figure 1. Workflow of the study design.

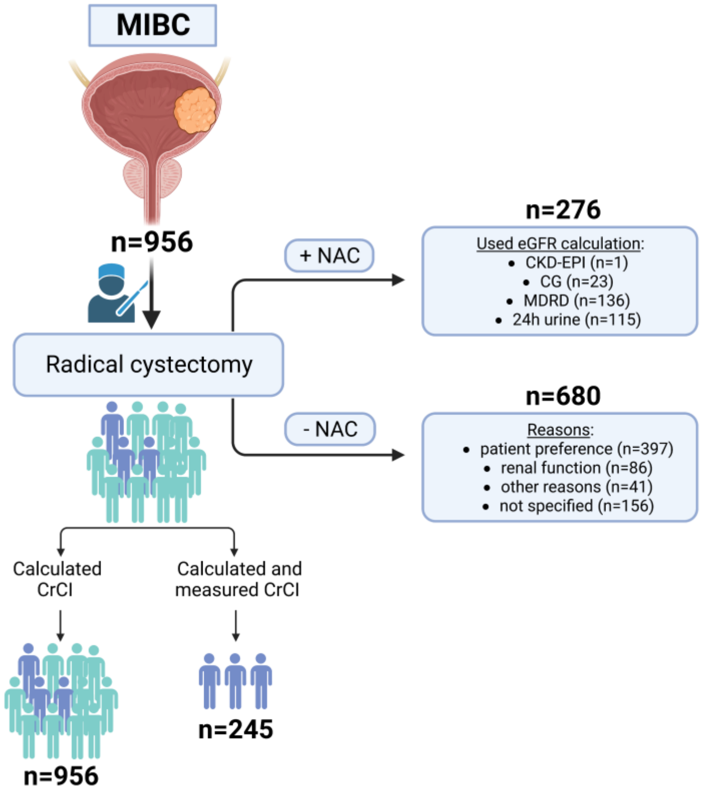

Suppl. Table 1. **Model equations for estimation of calculated eGFR and measured CrCl.**

Cockcroft-Gault
$eGFR = \frac{(140 - Age) x\ Weight\ (kg) x\ [0.85\ if\ female]}{72\ x\ sCr\ (\frac{mg}{dl})}$
**MDRD**
$eGFR = 175\ x\ sCr^{-1.154}\ x\ Age^{-0.203}\ x\ [1.212\ if\ black]\ x\ [0.742\ if\ female]$
**CKD-EPI 2009**
$eGFR = 141\ x\ \left[\min\left(\frac{sCr}{\kappa}, 1\right)^{\alpha}\ x\ max\left(\frac{sCr}{\kappa}, 1\right)^{-1.209}\right]\ x\ 0.993^{Age}\ x\ [1.018\ if\ female]\ x\ [1.157\ if\ black]$
$\alpha = -0.329\ for\ females;\ \alpha = -0.411\ for\ males$
$\kappa = 0.9\ for\ males;\ \kappa = 0.7\ for\ females$
**CKD-EPI race-free 2021**
$eGFR = 142\ x\ \left\vert\min\left(\frac{sCr}{\kappa}, 1\right)^{\alpha}\ x\ \max\left(\frac{sCr}{\kappa}, 1\right)^{-1.200}\right\vert\ x\ 0.9938^{Age}\ x\ [1.012\ if\ female]$
$\alpha = -0.241\ for\ females;\ \alpha = -0.302\ for\ males$
$\kappa = 0.9\ for\ males;\ \kappa = 0.7\ for\ females$
**Measured (urine) CrCl**
$CrCI = \frac{uCr\left(\frac{mg}{dl}\right) x\ Urine\ volume\ (ml)}{sCr\left(\frac{mg}{dl}\right) x\ collection\ time\ (\min)}$

Suppl. Table 2. Frequencies of measured CrCl <60 and ≥60 mL/min for calculated eGFR values <40, 40-59, and ≥60 mL/min, separately for CG, MDRD, CKD-EPI, and race-free CKD-EPI.

	**<40 mL/min**	**40-59 mL/min**	**≥60**
CG			
Measured CrCl	N=36	N=48	N=149
**<60 mL/min**	20 (55.6%)	6 (12.5%)	9 (6.0%)
**≥60 mL/min**	16 (44.4%)	42 (87.5%)	140 (94.0%)
MDRD			
Measured CrCl	N=28	N=65	N=152
**<60 mL/min**	20 (71.4%)	9 (13.8%)	8 (5.3%)
**≥60 mL/min**	8 (28.6%)	56 (86.2%)	144 (94.7%)
CKD-EPI			
Measured CrCl	N=30	N=55	N=160
**<60 mL/min**	20 (66.7%)	9 (16.4%)	8 (5.0%)
**≥60 mL/min**	10 (33.3%)	46 (83.6%)	152 (95.0%)
Race-free CKD-EPI			
Measured CrCl	N=24	N=50	N=171
**<60 mL/min**	18 (75.0%)	11 (22.0%)	8 (4.7%)
**≥60 mL/min**	6 (25.0%)	39 (78.0%)	163 (95.3%)

Suppl. Table 3. p-values of interaction for various clinical parameters of the probabilities of a measured CrCl value ≥60 mL/min, given a specific calculated eGFR value. Non-significant p-values mean that the probabilities are not significantly modified across various subgroups (values) of this parameter. Of note, analyses of ethnicity, ECOG, CCI, CHF, and NYHA are limited by low prevalence/variability in these variables. Abbreviations: BMI - body mass index; ECOG - Eastern Cooperative Oncology Group; CCI - Charlson Comorbidity Index; CHF - congestive heart failure; NYHA - New York Heart Association.

Parameters	CG	MDRD	CKD-EPI	CKD-EPI race-free
**Age**	0,512	0,350	0,717	0,646
**Sex**	0,946	0,552	0,622	0,631
**BMI**	**0,003**	0,555	0,575	0,592
**Ethnicity**	0,446	0,705	0,639	0,660
**ECOG**	0,444	0,303	0,358	0,352
**CCI**	0,310	0,302	0,358	0,358
**Smoking**	0,554	0,799	0,744	0,755
**CHF**	0,187	0,223	0,228	0,227
**Diabetes**	0,469	0,286	0,231	0,241
**NYHA**	0,881	0,729	0,742	0,746
